# Segmentectomy for cancer control in radiologically pure-solid clinical stage IA3 lung cancer

**DOI:** 10.1093/icvts/ivad138

**Published:** 2023-08-17

**Authors:** Atsushi Kamigaichi, Takahiro Mimae, Norifumi Tsubokawa, Yoshihiro Miyata, Hiroyuki Adachi, Yoshihisa Shimada, Hiroyuki Ito, Norihiko Ikeda, Morihito Okada

**Affiliations:** Department of Surgical Oncology, Hiroshima University, Hiroshima, Japan; Department of Surgical Oncology, Hiroshima University, Hiroshima, Japan; Department of Surgical Oncology, Hiroshima University, Hiroshima, Japan; Department of Surgical Oncology, Hiroshima University, Hiroshima, Japan; Department of Thoracic Surgery, Kanagawa Cancer Center, Yokohama, Japan; Department of Surgery, Tokyo Medical University, Tokyo, Japan; Department of Thoracic Surgery, Kanagawa Cancer Center, Yokohama, Japan; Department of Surgery, Tokyo Medical University, Tokyo, Japan; Department of Surgical Oncology, Hiroshima University, Hiroshima, Japan

**Keywords:** Non-small-cell lung cancer, Segmentectomy, Lobectomy, Pure-solid, Recurrence

## Abstract

**OBJECTIVES:**

This study aimed to compare cancer control after segmentectomy and lobectomy in patients with radiologically pure-solid clinical stage IA3 non-small-cell lung cancer (NSCLC).

**METHODS:**

Patients with radiologically pure-solid clinical stage IA3 NSCLC who underwent lobectomy or segmentectomy at 3 institutions between 2010 and 2019 were identified. We estimated propensity scores to adjust for confounding variables regarding tumour malignancy, including age, sex, smoking history, tumour size, maximum standardized uptake value on ^18^F-fluorodeoxyglucose positron emission tomography, lymph node dissection, histological type and lymphatic, vascular and pleural invasion. Cumulative incidence of recurrence (CIR) was evaluated as a primary end point.

**RESULTS:**

Among 412 patients, postoperative recurrence occurred in 7 of 44 patients (15.9%) undergoing segmentectomy, and 71 of 368 patients (19.3%) undergoing lobectomy. CIR was comparable between patients undergoing segmentectomy (5-year rate, 21.9%) and those undergoing lobectomy (5-year rate, 20.8%; *P *=* *0.88). Locoregional recurrence did not differ between patients undergoing segmentectomy (6.8%) and those undergoing lobectomy (9.0%). In multivariable analysis, segmentectomy (versus lobectomy) was not identified as an independent prognostic factor for CIR (hazard ratio, 1.045; 95% confidence interval, 0.475–2.298; *P *=* *0.91). In propensity score matching of 40 pairs, CIR was not significantly different between patients undergoing segmentectomy (5-year rate, 20.7%) and those undergoing lobectomy (5-year rate, 18.4%; *P *=* *0.81).

**CONCLUSIONS:**

Cancer control may be comparable between segmentectomy and lobectomy in patients with radiologically pure-solid clinical stage IA3 NSCLC. Further studies are warranted to clarify the survival benefits of segmentectomy in these patients.

**Figure:**
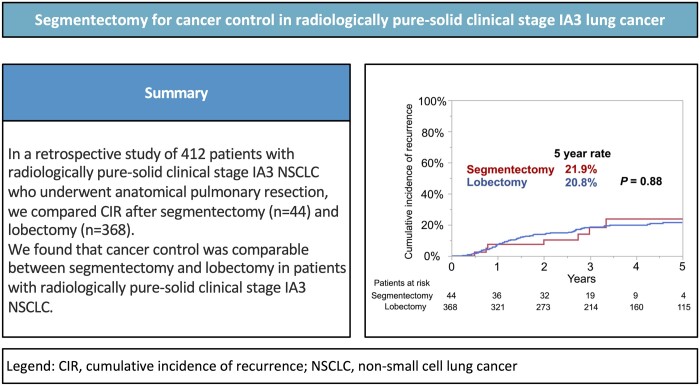

## INTRODUCTION

Lobectomy has been the recommended surgical procedure for early-stage non-small-cell lung cancer (NSCLC) for a long time after a randomized trial was published by the Lung Cancer Study Group [1]. However, recent clinical trials conducted by the Japanese Clinical Oncology Group (JCOG)/West Japan Oncology Group (WJOG) and the Cancer and Leukemia Group B, comparing the surgical results between lobectomy and sublobar resection for early-stage peripheral NSCLC, have shown the efficacy of sublobar resection in patients with early-stage lung cancer sized ≤2 cm [2, 3]. Furthermore, the JCOG1211 trial demonstrated the feasibility of segmentectomy even for radiologically ground-glass opacity (GGO)-predominant NSCLC up to 3 cm in maximum tumour size [4]. Based on these results, it is expected that an increasing number of patients with early-stage lung cancer will be treated with segmentectomy. In particular, the JCOG0802/WJOG4607L trial found that segmentectomy resulted in significantly better overall survival (OS) than lobectomy [2]. Therefore, it is expected that the range of applications of segmentectomy in early-stage NSCLC will widen in the future; the next target application of segmentectomy is in radiologically solid-predominant lung cancer sized >2–3 cm.

In a subgroup analysis of the JCOG0802/WJOG4607L trial, the benefit of segmentectomy in OS was observed to be higher in patients with pure-solid NSCLC than in those with part-solid NSCLC. However, lung cancer with a pure-solid appearance on high-resolution computed tomography (HRCT) shows higher malignancy compared with lung cancer with a GGO component [5, 6]. In addition, there are concerns regarding some disadvantages of segmentectomy, such as the risk of postoperative recurrence due to difficulties in ensuring adequate surgical margins and efficient assessment of lymph node metastasis compared with lobectomy, especially for larger and higher-grade tumours. Thus, the feasibility of segmentectomy, especially for patients with radiologically pure-solid NSCLC sized >2–3 cm (clinical stage IA3 NSCLC), remains controversial.

Therefore, this study aimed to compare cancer control after segmentectomy and lobectomy in patients with radiologically pure-solid clinical stage IA3 NSCLC.

## MATERIALS AND METHODS

### Ethics statement

This study was approved by the Institutional Review Boards of Hiroshima University Hospital (approval number E1216), Kanagawa Cancer Center (approval number 24-EKI-54) and Tokyo Medical University Hospital (approval number SH2969). The requirement for informed consent was waived because of the retrospective nature of the study.

### Study design and patient population

Data were obtained from consecutive patients diagnosed with clinical stage IA3 showing a pure-solid appearance on preoperative HRCT. These patients had undergone segmentectomy or lobectomy without induction therapy at the Hiroshima University Hospital, Kanagawa Cancer Center and Tokyo Medical University between January 2010 and December 2019, and the data were obtained from medical records and retrospectively analysed. The individual-level database was prospectively maintained. The preoperative staging was based on the findings of HRCT and 18-fluoro-2-deoxyglucose positron emission tomography/computed tomography (FDG-PET/CT). Preoperative endobronchial ultrasonography and mediastinoscopy had not been performed routinely. After excluding patients who had a tumour in the right middle lobe, the postoperative recurrence risk was compared with that after lobectomy (Supplementary Material, Fig. S1).

The tumour-node-metastasis classification of malignant tumours (8th edition) was used for tumour staging [7]. Pathological diagnosis was based on the World Health Organization Classification of Tumors of the Lung, Pleura, Thymus, and Heart [8]. Lymph node metastasis was considered negative when swollen mediastinal or hilar lymph nodes >1 cm in short axis diameter were not detected on HRCT and when there was no accumulation of FDG in these nodes on FDG-PET/CT images.

### High-resolution computed tomography and 18-fluoro-2-deoxyglucose positron emission tomography imaging

Chest images were acquired using a 16-row multidetector computed tomography (CT). High-resolution images were acquired using the following parameters: 120 kVp, 200 mA, section thickness of 2 mm, pixel resolution of 512 × 512, scan duration of 0.5–1.0 s, a high spatial reconstruction algorithm with a 20-cm field of view and mediastinal [window level, 40 Hounsfield units (HU); window width, 400–139 HU] and lung (window level, −600 HU; window width, 1600 HU) window settings. Radiologists from each participating institution reviewed all CT images and determined the tumour size.

For FDG-PET/CT, an anthropomorphic body phantom that conformed to the National Electrical Manufacturers Association standards was used to minimize the variability in the maximum standardized uptake value (SUV_max_), which could result from differences in preparation procedures, scan acquisition, image reconstruction and data analysis among the 3 study centres [9].

### Surgical procedure

Radical segmentectomy and lobectomy with lymph node dissection and sampling were performed using hybrid video-assisted thoracic surgery. The surgical approach and procedures were decided at a surgical conference based on the tumour location and patient status. In this study, segmentectomy was performed with a passive indication in a compromised patient who had comorbidities, low-performance status or low respiratory function. Intraoperatively, only suspicious lymph nodes or resection margins were assessed using frozen sections.

### Follow-up evaluation

All patients were followed up starting from the day of the surgery. Postoperatively, physical examination and chest radiography were performed every 3 months, and CT was performed every 6 months for the first 2 years. Thereafter, physical examination and chest radiography were performed every 6 months, and CT was performed annually. Recurrence was determined based on radiographic features or histological evidence. Local recurrence was defined as tumour recurrence in the preserved lobe. Regional recurrence was defined as tumour recurrence in the form of ipsilateral hilar or mediastinal lymph node metastasis. Other types of recurrence were defined as distant recurrence.

### Statistical analysis

Patient characteristics were reported as proportions or medians and interquartile ranges. Chi-squared and Wilcoxon rank-sum tests were used to compare the segmentectomy and lobectomy groups. McNemar’s test for categorical variables and paired *t*-test for continuous variables were used to analyse propensity-matched patient pairs.

Time-to-event end points were analysed using competing risk analysis. The risk of recurrence, defined as the cumulative incidence of recurrence (CIR), was estimated using a cumulative incidence function, which accounted for death without recurrence as a competing event. Patients were censored if they were alive and had no recurrence at the time of the last follow-up. Differences in CIR between the groups were assessed using the methods of Gray (in univariable non-parametric analyses) and Fine and Gray (in multivariable analyses). OS was defined as the time elapsed from surgery to death from any cause or censored at last alive follow-up. Recurrence-free survival (RFS) was defined as the time elapsed from the surgery to recurrence, death from any cause, or censored at last alive follow-up. Survival data were estimated using the Kaplan–Meier method and compared using the log-rank test. In the multivariable proportional hazards model, the variables included in the analysis were clinical variables such as age, sex, smoking history, solid tumour size, SUV_max_, surgical procedure, extent of lymph node dissection and histological type.

The propensity score was estimated using a logistic regression model based on factors related to tumour malignancy such as age (continuous), sex (female/male), smoking history (never/ever), tumour size (continuous), SUV_max_ (continuous), extent of lymph node dissection (ND1/ND2), histological type [adenocarcinoma (high grade/intermediate grade/low grade)/others], lymph vessel invasion (absent/present), vascular invasion (absent/present) and pleural invasion (absent/present) as explanatory variables. Greedy matching with a calliper width of 0.20 of the standard deviation of the logit transformation for the estimated propensity score was applied. Propensity score matching in a 1:1 ratio was performed using the estimated propensity score. Standardized differences were calculated to investigate the balance of patient characteristics.

Statistical significance was set at *P *<* *0.05. All statistical analyses were performed using JMP version 16 and EZR version 1.51 (Saitama Medical Center, Jichi Medical University, Saitama, Japan), which is a graphical user interface for R (The R Foundation for Statistical Computing, Vienna, Austria) [10].

## RESULTS

### Patient characteristics

Table 1 lists the characteristics of the 412 patients with radiologically pure-solid clinical stage IA3 NSCLC who underwent segmentectomy or lobectomy. Of these, 44 and 368 patients underwent segmentectomy and lobectomy, respectively. Significant differences in age (*P *<* *0.01), SUV_max_ (*P *<* *0.01), extent of lymph node dissection (*P *<* *0.01), number of resected lymph nodes and adjuvant therapy (*P *<* *0.01) were observed between the segmentectomy and lobectomy groups. In the segmentectomy group, 13 (29.5%) and 11 (25.0%) patients underwent left upper division and S6 segmentectomies, respectively (Supplementary Material, Table S1).

**Table 1:** Clinical characteristics of patients

Variables	Segmentectomy (*n* = 44)	Lobectomy (*n* = 368)	*P*-Value	SD
Age, median (IQR)	74 (70–78)	70 (63–76)	<0.001	0.527
Sex, male, *n* (%)	32 (72.7)	219 (59.1)	0.10	0.207
Smoking history, *n* (%)	34 (77.3)	252 (68.5)	0.30	0.121
Respiratory function,^a^ median (IQR)				
FEV, 1.0%	68.9 (48.0–75.2)	73.7 (68.1–79.5)	0.006	
VC, %	94.7 (92.0–111.9)	104.4 (92.6–119.0)	0.087	
Tumour location, upper lobe, *n* (%)	24 (54.6)	191 (51.9)	0.75	
SUV_max_, median (IQR)	4.0 (2.1–9.8)	6.0 (3.7–10.5)	0.015	−0.337
Tumour size (cm), median (IQR)	2.4 (2.2–2.8)	2.5 (2.2–2.7)	0.48	−0.107
Lymph node dissection, ND2, *n* (%)	23 (52.3)	337 (91.6)	<0.001	0.530
Histological type, *n* (%)				
Adenocarcinoma	27 (61.3)	265 (72.0)	0.14	0.161
High grade	4 (9.1)	16 (4.4)		
Intermediate	18 (40.9)	205 (55.7)		
Low grade	5 (11.4)	44 (12.0)		
Others	17 (38.6)	103 (28.0)		
Resected lymph nodes, median (IQR)	7 (4–11)	15 (10–21)	<0.001	
Pathological LN metastasis, *n* (%)				
N1	2 (4.6)	46 (12.5)	0.20	
N2	2 (4.6)	31 (8.4)		
Pleural invasion, *n* (%)	13 (29.6)	106 (28.8)	1.0	0.028
Lymph vessel invasion, *n* (%)	12 (27.3)	130 (35.3)	0.32	0.258
Vascular invasion, *n* (%)	21 (47.7)	195 (53.9)	0.53	0.123
Pathologically invasive cancer,^b^*n* (%)	27 (61.4)	248 (67.4)	0.50	
Adjuvant therapy, *n* (%)	4 (9.1)	134 (36.4)	<0.001	
Cause of death, *n* (%)				
Lung cancer	3 (6.8)	35 (9.5)	0.60	
Other cause	5 (11.4)	28 (7.6)		

a Reference values due to the unavailable data (24 in the segmentectomy group, 145 in the lobectomy group).

b Pathologically invasive cancer is defined as the presence of blood vessels, lymphatic and pleural invasion or nodal involvement.

FEV: forced expiratory volume; IQR: interquartile range; LN: lymph node; SD: standardized difference; SUV_max_: maximum standardised uptake value; VC: vital capacity.

### Cumulative risk of recurrence and survival outcomes

The median follow-up period after surgery was 47.1 (interquartile range, 30.4–64.6) months. Recurrence occurred in 7 (15.9%) patients in the segmentectomy group and 71 (19.3%) patients in the lobectomy group. The recurrence sites after segmentectomy and lobectomy are shown in Table 2. There was no difference in the recurrence pattern between the segmentectomy group (local: 0%, regional: 6.8%, distant: 11.4%) and the lobectomy group (local: 0.3%, regional: 8.7%, distant: 15.2%) (*P *=* *0.84).

**Table 2:** Relapse pattern and site after surgery

	Segmentectomy (*n* = 44), *n* (%)	Lobectomy (*n* = 368), *n* (%)
Total relapse	7 (15.9)	71 (19.3)
Local^a^	0	1 (0.3)
Surgical stump	0	1 (0.3)
Preserved lobe	0	0
Regional^a^	3 (6.8)	27 (7.3)
Hilar LN	1 (2.3)	8 (2.2)
Mediastinal LN	2 (4.5)	24 (6.5)
Distant^a^	5 (11.4)	56 (15.2)
Unknown	1	1

a Overlapping cases are included.

LN: lymph node.

**Figure 1:**
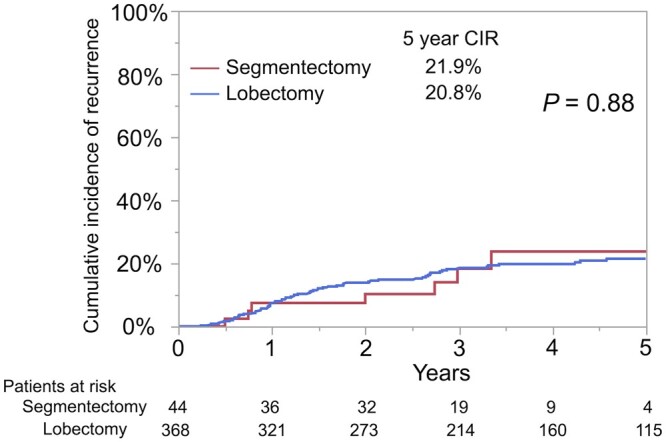
Cumulative incidence of recurrence for all patients. CIR was not significantly different between patients undergoing segmentectomy (5-year CIR, 21.9%; 95% CI, 9.2–38.2) and those undergoing lobectomy (5-year CIR, 20.8%; 95% CI, 16.4–25.5; *P* = 0.88). CI: confidence interval; CIR: cumulative incidence of recurrence.

Among all patients, the CIR did not differ between the segmentectomy group and the lobectomy group (5-year CIR: 21.9% vs 20.8%, *P *=* *0.88; Fig. 1). The results of univariable and multivariable analyses for CIR are presented in Table 3. The independent prognostic factor for CIR was increasing SUV_max_ (hazard ratio, 1.038; 95% confidence interval, 1.007–1.071; *P *=* *0.017) but not the surgical procedure (segmentectomy vs lobectomy) (hazard ratio, 1.045; 95% confidence interval, 0.475–2.298; *P *=* *0.91). OS and RFS were not significantly different between the groups (Supplementary Material, Fig. S2).

**Table 3:** Results of univariable and multivariable analysis of cumulative incidence of recurrence

Variables	Univariable			Multivariable		
	HR	95% CI	*P*-Value	HR	95% CI	*P*-Value
Age (+1 year)	0.988	0.967–1.008	0.24	0.990	0.967–1.013	0.39
Sex						
Female	Ref.			Ref.		
Male	0.597	0.370–0.964	0.035	0.746	0.429–1.296	0.30
Smoking history						
Never	Ref.			Ref.		
Ever	1.879	1.096–3.220	0.022	1.584	0.825–3.042	0.17
Tumour size, +1.0 mm	1.06	0.986–1.141	0.11	1.054	0.977–1.136	0.18
≤25 mm	Ref.					
>25 mm	1.133	0.725–1.771	0.58			
SUV_max_, +1.0	1.042	1.012–1.073	0.006	1.038	1.007–1.071	0.017
Location						
Lower	Ref.			Ref.		
Upper	1.138	0.729–1.777	0.57	0.939	0.583–1.513	0.80
Surgical procedure						
Lobectomy	Ref.					
Segmentectomy	0.925	0.430–1.989	0.84	1.045	0.475–2.298	0.91
Extent of lymph node dissection						
Hilar	Ref.			Ref.		
Hilar + mediastinum	1.585	0.683–3.68	0.28	1.374	0.579–3.263	0.47
Histological type						
Others	Ref.			Ref.		
Adenocarcinoma	0.847	0.526–1.364	0.49	1.324	0.760–2.307	0.32

CI: confidence interval; HR: hazard ratio; SUV_max_: maximum standardised uptake value.

### Cumulative risk of recurrence and survival outcomes among propensity score-matched pairs

**Figure 2:**
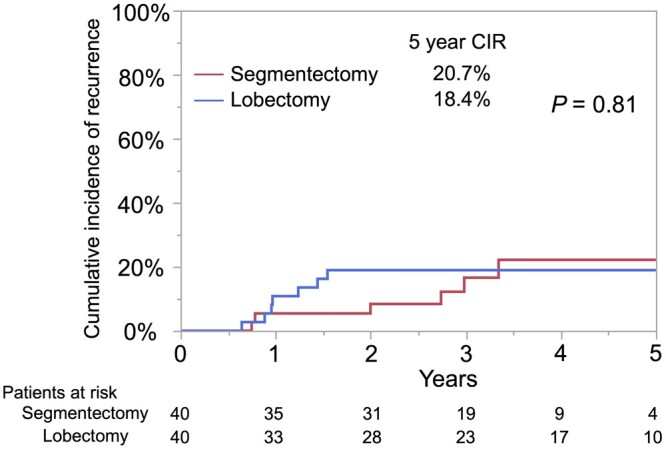
Cumulative incidence of recurrence for propensity score-matched pairs. CIR was not significantly different between patients undergoing segmentectomy (5-year CIR, 20.7%; 95% CI, 8.0–37.5) and those undergoing lobectomy (5-year CIR, 18.4%; 95% CI, 8.1–32.0; *P*=0.81). CI: confidence interval; CIR: cumulative incidence of recurrence.

The characteristics of propensity score matching are shown in Table 4. There were no significant differences in the factors related to tumour malignancy, including age, sex, smoking history, tumour size, SUV_max_, extent of lymph node dissection, histological type, lymph vessel invasion, vascular invasion and pleural invasion. Among the propensity score matching pairs, CIR was not different between the segmentectomy group and lobectomy group (5-year CIR: 20.7% vs 18.4%, *P *=* *0.81; Fig. 2). OS and RFS were not significantly different between the groups (Supplementary Material, Fig. S3).

**Table 4:** Characteristics of patients in propensity score-matched cohort

Variables	Segmentectomy (*n* = 40)	Lobectomy (*n* = 40)	*P*-Value	SD
Age, median (IQR)	74 (70–79)	76 (66–78)	0.71	0.123
Sex, male, *n* (%)	29 (72.5)	32 (80.0)	1.0	0.098
Smoking history, *n* (%)	31 (77.5)	32 (80.0)	1.0	0.032
Tumour location, upper lobe, *n* (%)	22 (55.0)	18 (45.0)	0.50	
SUV_max_, median (IQR)	4.1 (2.1–9.8)	4.8 (3.1–8.9)	0.47	−0.009
Tumour size (cm), median (IQR)	2.4 (2.2–2.8)	2.5 (2.3–2.7)	0.55	−0.110
Lymph node dissection, ND2, *n* (%)	23 (57.5)	23 (57.5)	1.0	0
Histological type, *n* (%)				
Adenocarcinoma	25 (62.5)	27 (67.5)	0.92	0.076
High grade	3 (7.5)	3 (7.5)		
Intermediate	17 (42.5)	17 (42.5)		
Low grade	5 (12.5)	7 (17.5)		
Others	15 (37.5)	13 (32.5)		
Resected lymph nodes, median (IQR)	7 (5–11)	12 (6–17)	0.029	
Pathological LN metastasis, *n* (%)				
N1	2 (5.0)	3 (7.5)	0.80	
N2	2 (5.0)	3 (7.5)		
Pleural invasion, *n* (%)	12 (30.0)	12 (30.0)	1.0	0
Lymph vessel invasion, *n* (%)	12 (30.0)	14 (35.0)	0.81	0.156
Vascular invasion, *n* (%)	17 (42.5)	21 (52.5)	0.50	0.211
Pathologically invasive cancer,^a^*n* (%)	23 (57.5)	24 (60.0)	1.0	
Adjuvant therapy, *n* (%)	4 (10.0)	13 (32.5)	0.027	

a Pathologically invasive cancer is defined as the presence of blood vessels, lymphatic and pleural invasion or nodal involvement.

IQR: interquartile range; SD: standardized difference.

## DISCUSSION

In this study, the postoperative recurrence risk was comparable between patients who underwent segmentectomy and lobectomy using multivariable and propensity score-matched analyses. We demonstrated acceptable cancer control with segmentectomy compared with lobectomy, even for radiologically pure-solid clinical stage IA3 NSCLC.

Cancer and Leukemia Group B/Alliance 14053 trial found that sublobar resection, including segmentectomy and wedge resection, was not inferior to lobectomy for disease-free survival [3]. In addition, the JCOG0802/WJOG4607L trial revealed the superiority of segmentectomy over lobectomy for OS and non-inferiority for RFS [2]. In both trials, the respiratory function was preserved better after sublobar resection than lobectomy. Reportedly, segmentectomy contributes to the preservation of postoperative respiratory function, nutritional status and reduces the risk of postoperative complications compared with lobectomy [2, 11–13]. Segmentectomy is considered to be a less invasive procedure than lobectomy with equivalent cancer curability for peripherally located early-stage NSCLC sized ≤2 cm. Furthermore, previous studies have reported the feasibility of segmentectomy, even for clinically aggressive or pathologically invasive NSCLC [14, 15]. Segmentectomy has a further confounding potential to provide favourable survival even for patients with larger and higher-grade NSCLC.

Pure-solid lung cancer without a GGO component on HRCT reportedly shows a worse prognosis than part-solid lung cancer with a GGO component [5, 6]. Although it is controversial whether a small GGO component has a favourable impact on tumour malignancy in lung cancer sized >2–3 cm, it is undoubtful that pure-solid lung cancer has highly malignant characteristics [16, 17]. Pure-solid lung cancer shows higher pathologic invasiveness, including lymphatic invasion, vascular invasion, lymph node metastasis and spread through air spaces (STAS) compared with lung cancer with a GGO [16–18]. The accuracy of lymph node dissection in sublobar resection is often debated, especially for lung cancer with a potentially high risk of unsuspected lymph node metastasis. In contrast, the JCOG0802/WJOG4607L trial found no difference in the frequency of nodal upstaging or hilar lymph node recurrence between segmentectomy and lobectomy [2]. In this study, the frequency of lymph node recurrence was comparable between the groups. This suggests that lymph node dissection could be performed adequately even with segmentectomy, although sites that are difficult to dissect by segmentectomy may exist. Furthermore, a recent large-scale retrospective study reported that there was no improvement in OS in patients undergoing lobectomy versus segmentectomy in patients with clinical stage IA NSCLC harbouring unexpected nodal disease [19]. Thus, segmentectomy may be an acceptable strategy even for patients with a high risk of unsuspected lymph node metastasis. In addition, the risk of lymph node metastasis is reported to depend on tumour location, whether central or peripheral, rather than the malignancy of the tumour itself [20, 21]. Thus, NSCLC located in the periphery of the lung field had a lower frequency of lymph node metastasis than that located in central lesions, and the risk of unsuspected lymph node metastasis was comparable between <2 and 2–3 cm peripheral pure-solid lung cancer [20, 21].

STAS was observed in 22% of patients with pure-solid lung cancer sized >2–3 cm, which could be a risk factor for marginal recurrence after segmentectomy. However, in previous reports, the recurrence risk was similar between segmentectomy and lobectomy for NSCLC with STAS, if the surgical margin was adequate [22]. Based on the previous reports, a surgical margin ≥20 mm could prevent postoperative recurrence even for pure-solid lung cancer sized >2–3 cm [23]. In this study, no local recurrence was observed in the segmentectomy group. Pure-solid tumours are more easily and accurately palpable than part-solid tumours, which may provide adequate surgical margins and contribute to the low recurrence risk, although this study did not evaluate the surgical margin.

This study focused only on radiologically pure-solid clinical stage IA3 lung cancer and evaluated postoperative recurrence risk. Meanwhile, some previous studies have reported the feasibility of segmentectomy for patients with lung cancer sized >2–3 cm which was considered to include mainly solid predominat tumors [11, 24–28], except for some reports that did not adjust for patients’ backgrounds [29, 30]. In a recent retrospective study investigating the prognostic validity of sublobar resection, including segmentectomy and wedge resection, using the Japanese Joint Committee of Lung Cancer Registry Database, segmentectomy tended to have worse OS (*P *=* *0.077) and disease-free survival (*P *=* *0.39) than lobectomy in patients with clinical stage IA3 pure-solid NSCLC, albeit not significantly [24]. However, in the multivariable analysis adjusted for patient background, such as performance status, comorbidities and respiratory function, segmentectomy had comparable survival outcomes compared with other surgical procedures, including lobectomy (lobectomy, 93.1%; wedge resection, 6.9%). Certainly, patients should be carefully selected because of the higher risk of local relapse in patients undergoing segmentectomy than in those undergoing lobectomy based on the evidence from the JCOG0802/WJOG4607L trial. However, referring to the results of previous studies and our study, segmentectomy could achieve curative resection and acceptable survival for radiologically pure-solid lung cancer sized >2–3 cm.

### Limitations

This study had several limitations. First, this was a retrospective analysis of prospectively collected clinicopathological data from patients at 3 institutions. The unresolved heterogeneity of patient backgrounds, including respiratory function, performance status, comorbid conditions and unobserved potential confounders, could be problematic, especially in multicentre retrospective studies. In addition, segmentectomy was performed for compromised patients with passive intent, even though there were no clear criteria. Therefore, comparison of survival outcomes, including OS and RFS, was difficult. Therefore, we evaluated CIR as a main outcome, because it reflects cancer control by the surgical procedure itself. Second, mediastinal lymph node dissection was performed in 52.3% of the segmentectomy group and 91.6% in the lobectomy group due to surgery with passive indication, which is not in line with the current recommendation. This might affect the difference in upstaging rate between the groups and have an impact on survival outcomes. Third, this study was underpowered to answer the study questions owing to the small number of cases, especially in the segmentectomy group. Fourth, the median follow-up duration of 47.1 months in this study might have been too short to accurately evaluate late recurrence.

## CONCLUSION

Although segmentectomy was performed with passive indication and there were unobserved potential confounders, cancer control might be comparable between segmentectomy and lobectomy in patients with radiologically pure-solid clinical stage IA3 NSCLC. Further studies are warranted for conclusive judgement of the benefits of segmentectomy for patients with radiologically pure-solid clinical stage IA3 NSCLC.

## Supplementary Material

ivad138_Supplementary_Data

## Data Availability

The data underlying this study are available in this article and in the online supplementary material.

## ABBREVIATIONS

CIR: Cumulative incidence of recurrence
CT: Computed tomography
FDG-PET/CT: 18-Fluoro-2-deoxyglucose positron emission tomography/computed tomography
GGO: Ground-glass opacity
HRCT: High-resolution computed tomography
HU: Hounsfield units
JCOG: Japanese Clinical Oncology Group
NSCLC: Non-small-cell lung cancer
OS: Overall survival
RFS: Recurrence-free survival
STAS: Spread through air spaces
SUV_max_: Maximum standardized uptake value
WJOG: West Japan Oncology Group
