# Supplementary material for: Segmentectomy for cancer control in radiologically pure-solid clinical stage IA3 lung cancer
Source: Interdiscip Cardiovasc Thorac Surg. 2023 Aug 17;37(3):ivad138. doi: 10.1093/icvts/ivad138 (PMC10533752; doi:10.1093/icvts/ivad138)
Supplement: ivad138_Supplementary_Data [file ivad138_supplementary_data.zip › Supplementary Table S1.docx]

Supplementary Table S1. Resected segment in patients undergoing segmentectomy

| Right | | Left | |
| --- | --- | --- | --- |
| Right upper lobe | 4 (9.1%) | Left upper lobe | 20 (45.5%) |
| S1a+2 | 1 (2.3%) | S1+2 | 2 (4.5%) |
| S2 | 2 (4.5%) | S1+2+3 | 13 (29.5%) |
| S3 | 1 (2.3%) | S3 | 2 (4.5%) |
|  |  | S4+5 | 3 (6.8%) |
| Right lower lobe | 12 (27.3%) | Left lower lobe | 8 (18.2%) |
| S6 | 6 (13.6%) | S6 | 5 (11.4%) |
| S8 | 1 (2.3%) | S8+9+10 | 2 (4.5%) |
| S9 | 2 (4.5%) | S9+10 | 1 (2.3%) |
| S9+10 | 1 (2.3%) |  |  |
| S10 | 1 (2.3%) |  |  |
| S7+8+9+10 | 1 (2.3%) |  |  |
