# Supplementary material for: Segmentectomy for cancer control in radiologically pure-solid clinical stage IA3 lung cancer
Source: Interdiscip Cardiovasc Thorac Surg. 2023 Aug 17;37(3):ivad138. doi: 10.1093/icvts/ivad138 (PMC10533752; doi:10.1093/icvts/ivad138)
Supplement: ivad138_Supplementary_Data [file ivad138_supplementary_data.zip › Supplementary_Figure_legend.docx]

Supplementary Figure 1. Patient selection flowchart

Preoperative staging was based on the TNM Classification of Malignant Tumors, 8th Edition.

Abbreviation: TNM, Tumour–Node–Metastasis

Supplementary Figure 2. Overall survival (A) and recurrence-free survival (B) curves between patients undergoing segmentectomy and those undergoing lobectomy in all patients.

Supplementary Figure 3. Overall survival (A) and recurrence-free survival (B) curves between patients undergoing segmentectomy and those undergoing lobectomy in propensity-score matched pairs.
