# Supplementary figures and images for: Segmentectomy for cancer control in radiologically pure-solid clinical stage IA3 lung cancer
Source: Interdiscip Cardiovasc Thorac Surg. 2023 Aug 17;37(3):ivad138. doi: 10.1093/icvts/ivad138 (PMC10533752; doi:10.1093/icvts/ivad138)

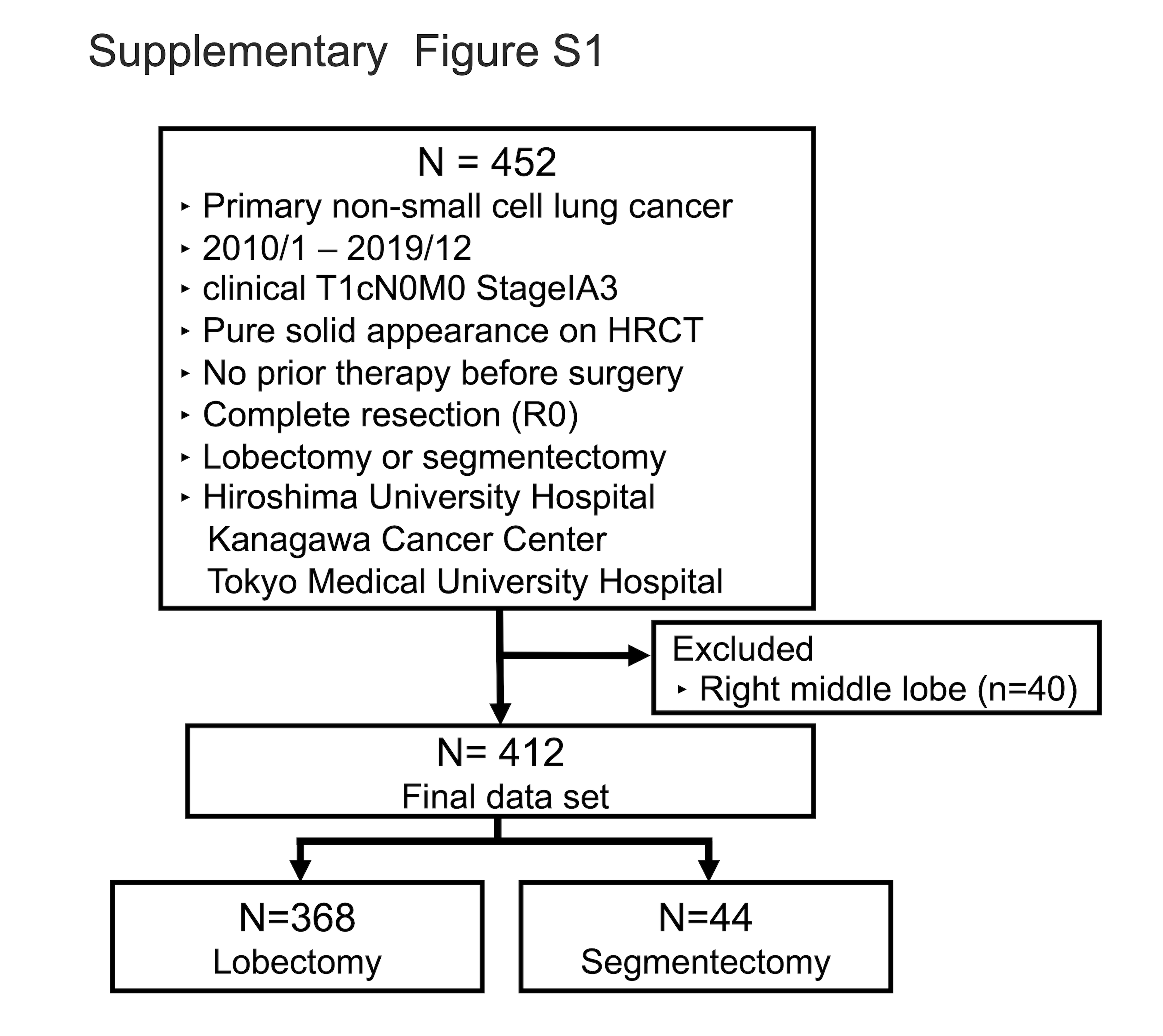

Supplement: ivad138_Supplementary_Data [file ivad138_supplementary_data.zip › Supplementary Figure S1.tiff]

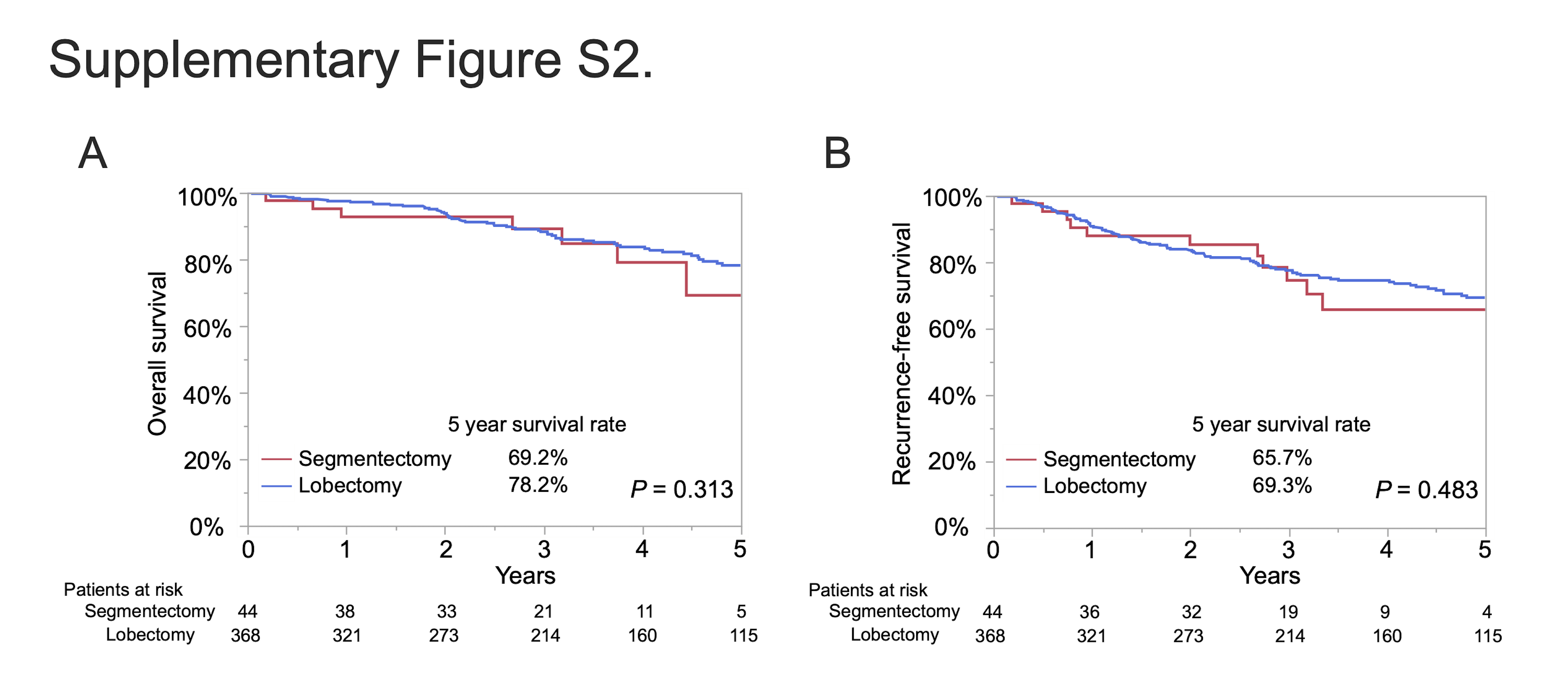

Supplement: ivad138_Supplementary_Data [file ivad138_supplementary_data.zip › Supplementary Figure S2.tiff]

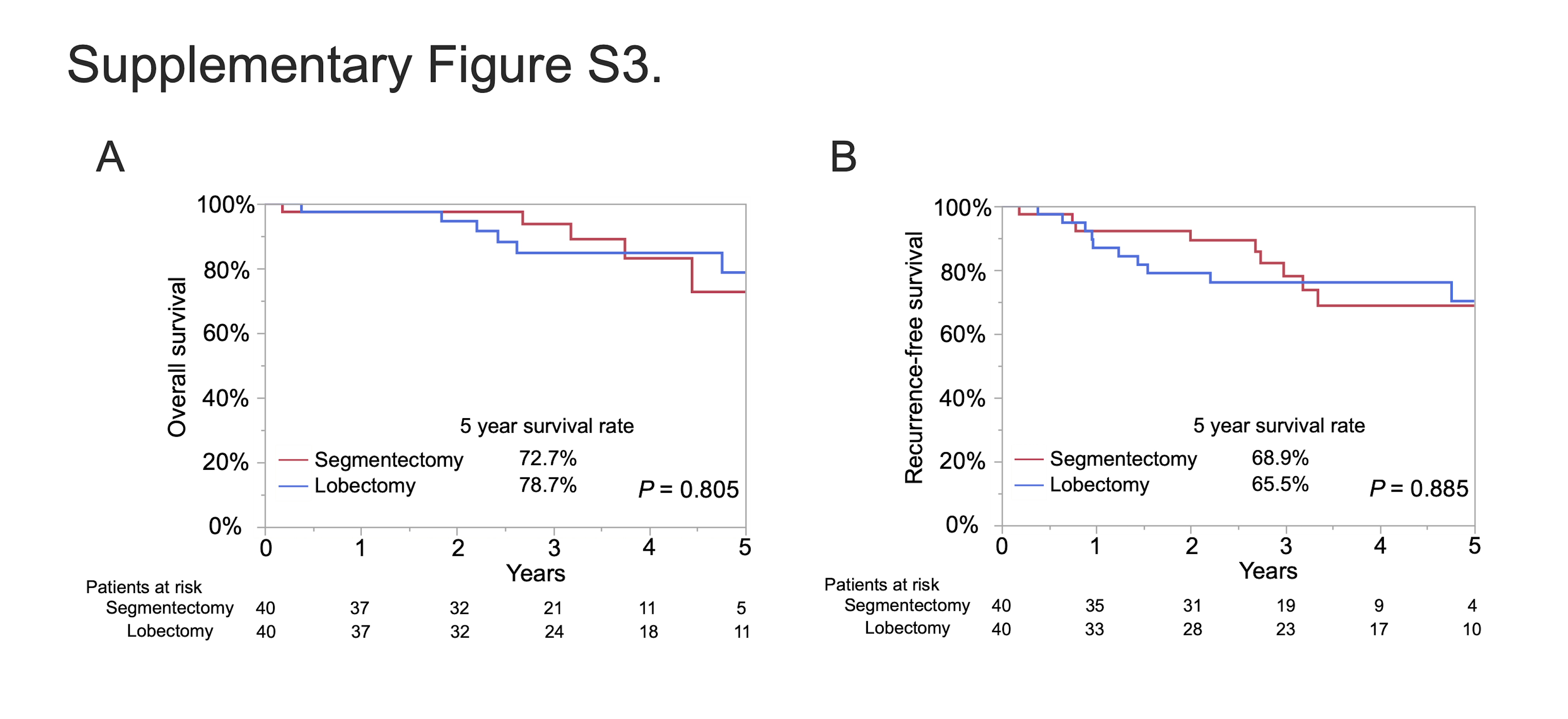

Supplement: ivad138_Supplementary_Data [file ivad138_supplementary_data.zip › Supplementary Figure S3.tiff]
